# Coinhibitory Receptor Expression and Immune Checkpoint Blockade: Maintaining a Balance in CD8^+^ T Cell Responses to Chronic Viral Infections and Cancer

**DOI:** 10.3389/fimmu.2017.01215

**Published:** 2017-09-29

**Authors:** Isobel S. Okoye, Michael Houghton, Lorne Tyrrell, Khaled Barakat, Shokrollah Elahi

**Affiliations:** ^1^Department of Dentistry, University of Alberta, Edmonton, AB, Canada; ^2^Department of Medical Microbiology and Immunology, University of Alberta, Edmonton, AB, Canada; ^3^Faculty of Medicine and Dentistry, Li Ka Shing Institute of Virology, University of Alberta, Edmonton, AB, Canada; ^4^Faculty of Pharmacy and Pharmaceutical Sciences, University of Alberta, Edmonton, AB, Canada

**Keywords:** T cell exhaustion, immune checkpoints, chronic infections, cancer, checkpoint blockade, immunotherapy

## Abstract

In cancer and chronic viral infections, T cells are exposed to persistent antigen stimulation. This results in expression of multiple inhibitory receptors also called “immune checkpoints” by T cells. Although these inhibitory receptors under normal conditions maintain self-tolerance and prevent immunopathology, their sustained expression deteriorates T cell function: a phenomenon called exhaustion. Recent advances in cancer immunotherapy involve blockade of cytotoxic T lymphocyte antigen-4 and programmed cell death 1 in order to reverse T cell exhaustion and reinvigorate immunity, which has translated to dramatic clinical remission in many cases of metastatic melanoma and lung cancer. With the paucity of therapeutic vaccines against chronic infections such as HIV, HPV, hepatitis B, and hepatitis C, such adjunct checkpoint blockade strategies are required including the blockade of other inhibitory receptors such as T cell immunoreceptor with immunoglobulin (Ig) and immunoreceptor tyrosine-based inhibitory motif domains, T cell Ig and mucin-domain containing-3, lymphocyte activation gene 3, and V-domain Ig-containing suppressor of T cell activation. The nature of different chronic viral infections and cancers is likely to influence the level, composition, and pattern of inhibitory receptors expressed by responding T cells. This will have implications for checkpoint antibody blockade strategies employed for treating tumors and chronic viral infections. Here, we review recent advances that provide a clearer insight into the role of coinhibitory receptor expression in T cell exhaustion and reveal novel antibody-blockade therapeutic targets for chronic viral infections and cancer. Understanding the mechanism of T cell exhaustion in response to chronic virus infections and cancer as well as the nature of restored T cell responses will contribute to further improvement of immune checkpoint blockade strategies.

## Introduction

The mammalian immune system functions to protect the host from challenge by a milieu of foreign antigens as well as prevention of autoimmunity by limiting recognition of self-antigens. The span of activity of the host defense system ranges from innate responses that respond within hours to the adaptive response specialized to recognize specific antigens and resolve infections.

T cells or lymphocytes are major components of the adaptive immune system consisting of CD4^+^ helper T cells and cytotoxic CD8^+^ T cells. Both subsets possess an antigen-recognition T cell receptor (TCR) generated by genetic recombination, which promotes the diversity of T cell responses (1). CD4^+^ and CD8^+^ T cells elicited in response to microbial invasion or tumor growth are subject to immune control to avert undue immunopathology and autoimmunity (2–8). Several arms of immune regulation are involved, including thymus-derived natural as well as inducible regulatory T cells (Tregs), and inhibitory receptors such as programmed cell death 1 (PD-1) and cytotoxic T lymphocyte antigen-4 (CTLA-4) (2, 9, 10).

Effector CD8^+^ T cells resolve acute viral infections within 5–7 days by producing cytokines and cytotoxic mediators followed by cell contraction and persistence of the memory pool [reviewed in Ref. (11)]. However, upon persistent exposure to antigen, which occurs during chronic infections, CD8^+^ T cells undergo a hierarchical loss of function and an associated increase in the expression of coinhibitory receptors. This phenomenon, termed exhaustion, is also exhibited by tumor-specific CD8^+^ T cells present in the tumor microenvironment. Several features make up the exhaustion phenotype. Exhausted cytotoxic T lymphocytes (CTLs) lose robust effector functions such as their ability to produce cytokines, capacity to proliferate, cytotoxicity required for killing virus-infected and tumor cells, and effective memory cell generation (12–14). Instead they express multiple coinhibitory receptors (also referred to as “immune checkpoints”) that render patients unable to mount an effective CTL response against tumors and chronic viral infections (9, 15). Exhaustion is also graded according to the number of coinhibitory receptors coexpressed by affected cells (16), and coinhibitory receptors such as PD-1 have been shown to be more indicative of this state compared to others (17). Importantly targeting coinhibitory receptors such as PD-1 and CTLA-4 using monoclonal antibodies, alone or in combination, has proven to be effective in restoring the function of exhausted T cells (18).

The onset of T cell exhaustion is highly dependent on persistent exposure of T cells to a wide range of antigens and stimuli derived from viruses, tumor antigens, and damage-associated molecular patterns (14, 19, 20). Consequently, the phenotypic and functional nature of exhausted T cells may vary according to the nature of eliciting antigens. Here, we focus on how coinhibitory receptor coexpression by CD8^+^ T cells defines T cell exhaustion in various chronic viral infections and cancer based on T cell function, disease severity, and effectiveness of immune checkpoint blockade.

## The CD8^+^ T Cell Response

### The Acute CD8^+^ T Cell Response

Major histocompatibility complex I-restricted CD8^+^ T cells are a major component of adaptive immune responses to intracellular pathogens and tumors. During acute immune responses to viruses such as lymphocytic choriomeningitis virus (LCMV) Armstrong or self-tumor antigens, naive CD8^+^ T cells undergo rapid proliferation and differentiation upon recognition of their cognate antigen. Like their “innate counterparts,” the natural killer cells, they “kill” by undergoing degranulation and secreting cytotoxic mediators such as perforin and granzymes (21). This is associated with other hallmarks of the effector response such as proliferation and production of IFN-γ, interleukin (IL)-2, and TNF-α, required to clear pathogens and resolve infections (22, 23).

Effector CD8^+^ T cells are characterized using various cell surface markers, which indicate differentiation stage (CCR7, CD62L, CD45RO, CD45RA, CD27, CD28, KLRG1), tissue localization (CD103), and memory differentiation potential (IL-7R and CD62L) (23, 24). Furthermore, they express multiple inhibitory receptors upon activation such as PD-1, T cell immunoglobulin (Ig) and mucin-domain containing-3 (TIM-3), CTLA-4, lymphocyte activation gene 3 (LAG-3), CD160, BTLA, V-domain Ig-containing suppressor of T cell activation (VISTA), and 2B4 [reviewed in Ref. (25)], which are required to control excessive inflammation and immunopathology.

Upon antigen clearance, approximately 90% of effector CD8^+^ T cells die by activation-induced cell death while the remaining 10% persist as memory cells, capable of undergoing IL-7- and IL-15-dependent homeostatic proliferation (26, 27). In the event of antigen re-challenge memory CD8^+^ T cells are able to mount a more rapid and robust response compared to primary responses mediated first by highly cytotoxic effector memory CD8^+^ T cells and subsequently central memory T cells with high proliferative ability (27).

### Chronic CD8^+^ T Cell Responses and T Cell Exhaustion

**Figure 1:**
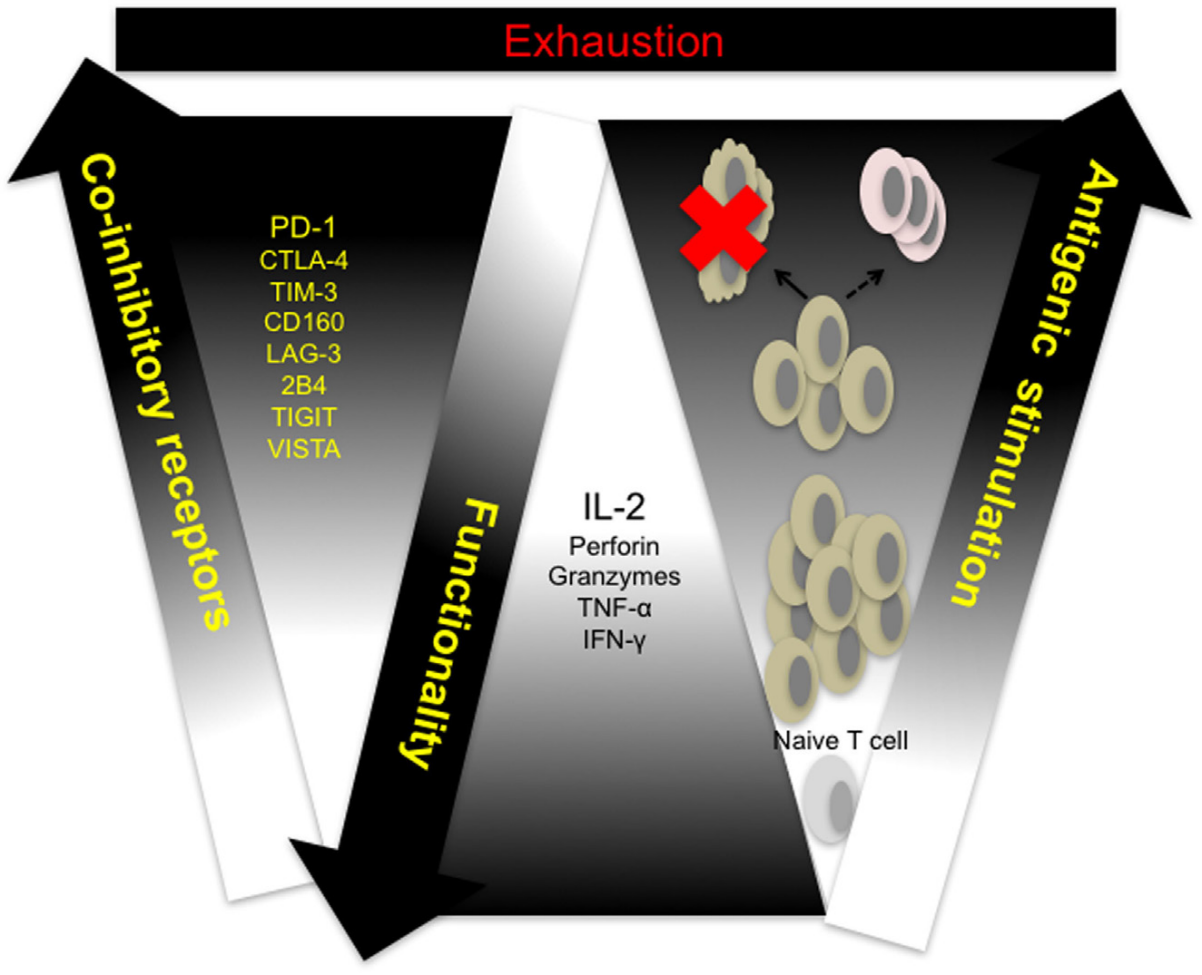
T cell exhaustion: a hierarchical loss of T cell function. Naive T cells differentiate and proliferate into effector cells in response to antigenic challenge. Sustained antigen exposure and T cell receptor (TCR) signaling in response to viral growth or tumor development results in progressive loss of function and concomitant upregulation of multiple coinhibitory receptors by responding cells. Responding T cells either undergo activation-induced cell death (clonal deletion) or exhaustion resulting in compromised memory T cell generation. CTLA-4, cytotoxic T-lymphocyte-associated protein 4; IFN-γ, interferon-gamma; IL-2, interleukin-2; LAG-3, lymphocyte-associated gene 3; PD-1, programmed cell death 1; PD-L1, programmed cell death ligand 1; TIGIT, T cell immunoreceptor with immunoglobulin (Ig) and immunoreceptor tyrosine-based inhibitory motif (ITIM) domains; TIM-3, T cell immunoglobulin and mucin domain containing-3, TNF-α, tumor necrosis factor alpha; VISTA, V-domain Ig-containing suppressor of T cell activation.

Cytotoxicity and other effector CD8^+^ T cell functions are compromised in response to sustained antigen exposure seen in chronic viral infections and in the peripheral blood and tumor microenvironment of cancer patients. This phenomenon, termed exhaustion, is described as a hierarchical loss of function by CD8^+^ T cells, starting with diminished production of effector cytokines, mainly IL-2, reduced proliferation and cytotoxicity, defective memory cell generation, culminating in deletion of affected cells (Figure 1) (28). Persistent antigenic stimulation and subsequent differentiation are prerequisites for T cell exhaustion unlike anergy which occurs when naive T cells undergo weak priming due to the absence of costimulation and signaling from inflammatory cytokines (29, 30). Recent studies utilizing novel molecular biology, transcriptional approaches, and analytical tools have shown that CTL exhaustion is a complex occurrence, involving changes in cell metabolism, transcriptional and posttranscriptional regulation (31–36). In a recent study, results from ATAC-seq of LCMV clone 13 CD8^+^ T cells showed that the chromatin-accessible regions of exhausted CD8^+^ T cells are distinct from non-exhausted cells and are adjacent to genes such as *PCDC1* (PD-1) (36). This observation suggests that exhausted T cells are a distinct lineage—restoration of function dependent on the level of antigenic stimulation. Indeed, the fixed genetic landscape of exhausted CD8^+^ T cells is apparent in reversion to exhaustion upon cessation of programmed cell death ligand 1 (PD-L1) blockade treatment (37).

Despite its multifaceted nature, CTL exhaustion has been primarily characterized by phenotypic expression of multiple coinhibitory receptors such as PD-1, CTLA-4, LAG-3, TIM-3, T cell immunoreceptor with Ig and immunoreceptor tyrosine-based inhibitory motif (ITIM) domains (TIGIT), VISTA, BTLA, 2B4, and CD160 by antigen-specific T cells (16). Coinhibitory receptors are a heterogeneous family of molecules that mediate negative regulation through a variety of ways, ranging from sequestration of costimulatory receptor ligands, upregulation of inhibitory genes to employing inhibitory sequence motifs such as ITIMs and ITSMs (15, 38). Understanding the relative contribution of individual coinhibitory receptors in promoting defective T cell responses will facilitate the development of more precise checkpoint blockade strategies.

The expression of coinhibitory receptors in addition to a milieu of signals intrinsic to CD8^+^ T cells and their microenvironment synergize to counter subsequent cell proliferation, acquisition of effector properties, and memory generation [reviewed in Ref. (13)]. Upregulation and sustained coexpression of coinhibitory receptors is regarded as the hallmark of CTL exhaustion; immune checkpoint blockade targeting CTLA-4 and/or PD-1/PD-L1 has achieved considerable success in the treatment of melanoma and other cancers (39–42). Furthermore, antibody blockade treatments targeting CTLA-4 and PD-1 in HIV and hepatitis B and C patients have been described (43–47).

In order to increase our understanding of T cell dysfunction and facilitate current checkpoint blockade interventions, there is need to differentiate the upregulation of coinhibitory receptors observed in response to T cell activation from exhaustion-based coinhibitory receptor expression. In two recent studies distinct gene modules that differentiate T cell dysfunction from activation were identified (48, 49). Singer et al. using a mouse CT26 colon carcinoma model, have shown that the zinc regulators, metallothioniens, promote tumor growth (48). They further demonstrated that CD8^+^ T cells from mice deficient in metallothioniens could not be differentiated from wild-type cells based on coexpression of TIM-3 and PD-1 alone. In a series of elegant gene-profiling experiments and principle component analyses, they identified gene modules for T cell dysfunction, which included known coinhibitory receptors (PD-1, CTLA-4, LAG-3, TIM-3, TIGIT) as well as costimulatory receptors of the TNF receptor family (TNFRSF4, TNFRSF9, and TNFRSF18) (48). Remarkably, results from this study correlated with the observations of Tirosh et al. who carried out single cell RNA sequencing of CD8^+^ T cells from melanoma tumors and were able to identify high and low exhaustion profiles relative to expression of cytotoxicity genes (49). High exhaustion genes included TNFRSF1B, TNFRSF9, and TIGIT. In addition NFATC1 and coinhibitory receptors such as TIM-3, PD-1, CTLA-4, and LAG-3 were variably expressed in tumors analyzed (49). These results indicate that expression of coinhibitory receptors and regulatory-associated TNF receptors identify exhausted T cells in cancer and possibly chronic viral infections. However, the existence of unique signatures associated with exhausted T cells from different settings is highly likely. Results from a study by Schietinger et al. based on a tamoxifen-inducible liver cancer mouse model indicate that although day 8 and day 30 TCR_SV40-I_-specific CD8^+^ T cells expressed similar exhaustion genes compared to LCMV clone 13 CD8^+^ T cells, distinct signatures were associated with each setting (50). Interestingly, *EOMES*, which has been shown to be associated with progressive exhaustion CD8^+^ T cells in chronic LCMV infection, was not expressed by dysfunctional TCR_SV40-I_-specific CD8+ T cells (50). The data on the role of coinhibitory receptors in T cell exhaustion indicate that these pathways are non-redundant. These coinhibitory molecules are originated from different structural families, bind ligands with unique expression patterns and possess well defined intracellular signaling domains. Thus, T cells from different chronic settings need to be extensively characterized using phenotypic, functional, and genomic approaches.

### Is PD-1 the Master Driver of T Cell Exhaustion?

The expression and progressive upregulation of PD-1 by virus and tumor-specific CD8^+^ T cells has been extensively studied and is regarded as the main indicator of CTL exhaustion in various chronic virus infections and tumor studies. Ubiquitous expression of PD-1 by exhausted T cells is possibly linked to its ability to mediate T cell suppression by multiple mechanisms [including induction of inhibitory genes such as BATF (51), targeting CD28 (52), recruitment of the intracellular phosphatases Src-homology domain-containing phosphatase (SHP) 1 and SHP2 to facilitate dephosphorylation of molecules downstream of the TCR and suppression of the transcription factor SKP2 (53)]. In addition, PD-1 expressed by B cells and myeloid cells may synergize with T cell-intrinsic PD-1 in chronic infection and cancer scenarios (54–57).

The restoration of immune functions in exhausted CD8^+^ and CD4^+^ T cells as a consequence of PD-1/PD-L1 blockade has been demonstrated (17, 18, 58). Furthermore, a number of immune-checkpoint blockade studies have demonstrated that targeting a combination of PD-1 and other coinhibitory receptors such as CTLA-4, TIM-3, CD160, TIGIT, and LAG-3 have facilitated restoration of CD8^+^ T cell function depicted by increased proliferation, IFN-γ and IL-2 production, and cytotoxicity (59–63). It is unclear, however, whether blockade of PD-1 is a major prerequisite for reversal of exhaustion. Selective expression of PD-1 or expression at higher levels by exhausted T cells compared to other inhibitory receptors could be contributory factors. Multiple coinhibitory receptors including PD-1 are upregulated and coexpressed by exhausted CD8^+^ T cells; however, the relative abundance of cells that express only PD-1 tends to exceed T cells that express other coinhibitory receptors (16).

The significance of PD-1 expression by exhausted CD8^+^ T cells is portrayed by its coordinated regulation by multiple transcription factors in order to contain increased antigenic stimulation. For instance, direct repression of PD-1 by T-bet in order to maintain virus-specific responses has been observed after day 15 of mouse LCMV clone 13 infection (64). This is dependent on the level of antigen exposure as increased CD8^+^ T cell stimulation corresponded with reduction in T-bet expression (64). In a recent study, PD-1 has also been shown to be regulated by glycogen synthase kinase 3 (GSK-3) (65). The targeting of GSK-3 using inhibitors, led to a reduction in PD-1 expression and clearance of LCMV clone 13 and reversal of T cell exhaustion (65). Interestingly, inactivation of GSK-3 was found to increase transcription of TBX21, the gene that encodes T-bet (65). Increased antigenic stimulation has been shown to impair activation of AKT (protein kinase B) and mechanistic target of rapamycin (mTOR) in exhausted CD8^+^ T cells with a concomitant increase in FoxO1 (32). FoxO1 promotes the sustenance of PD-1^+^ exhausted CD8^+^ T cells and the control of chronic LCMV infection (32). These studies highlight measures in place, based on regulation of PD-1 expression, to promote CTL responses during chronicity.

**Figure 2:**
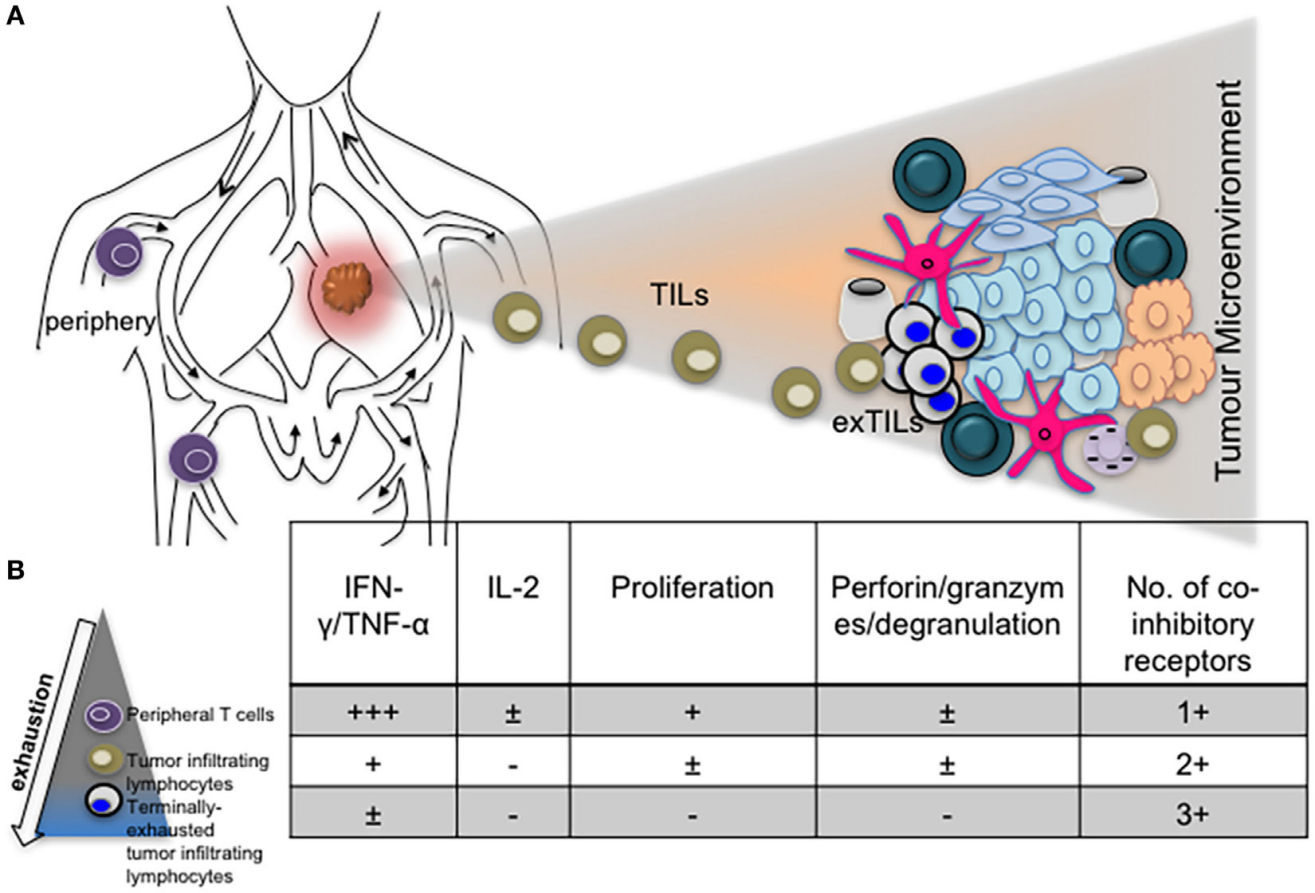
The phenotype and function of exhausted T cells may be influenced by anatomical location. **(A)** Diagram depicting T cells in the periphery and lung tumor microenvironment. **(B)** Progressive exhaustion of T cells occurs with proximity to disease site. Table showing levels of cytokine expression, cytotoxicity, and number of coinhibitory receptors expressed by peripheral, tumor infiltrating and tissue resident T cells.

The role of other inhibitory receptors in regulating CTL responses during chronic virus infections however cannot be ruled out. Other transcriptional regulators such as Blimp-1 have been shown to regulate expression of PD-1 as well as other inhibitory receptors by CD8^+^ T cells in order to maintain a balance between effector function and exhaustion (31). T-bet also regulates the expression of LAG-3, CD160, BTLA and to a lesser extent, TIM-3 and 2B4, expressed by CD8^+^ T cells during clone 13 infection (64). Differential expression of coinhibitory receptors by exhausted T cells in various infections, different stages of infection/exhaustion, anatomical locations, tumor microenvironment, and in response to antiviral treatment is well documented (66–69) (Figure 2).

Selective, sustained expression, and transcriptional regulation of PD-1 may be due to its role in fine-tuning the balance between T cell exhaustion and activation. In a study on the development of PD-1-deficient CD8^+^ T cell responses in the absence of immunopathology, the absence of PD-1 was insufficient to prevent the development of exhaustion (70). However, absence of PD-1 disrupted the balance between T-bet^hi^ and EOMES^hi^ exhausted CD8^+^ T cells, leading to increased frequency of terminally exhausted antigen-specific CD8^+^ T cells. This observation is consistent with the finding that antibody-blockade of PD-1 or PD-L1 elicits two groups of exhausted CD8^+^ T cells with either restored functionality (T-bet^hi^ EOMES^lo^) or terminally exhausted (T-bet^lo^ EOMES^hi^) (64, 71–73). It is likely that genetic ablation of PD-1 favors generation of terminally exhausted CD8^+^ T cells (70). This is not surprising, as studies have demonstrated compensatory upregulation of other inhibitory receptors in response to PD-1 blockade (74, 75).

These observations indicate that PD-1 does not drive exhaustion but regulates responses of PD-1^+^ T cells during chronic infections. PD-1 utilizes a two-pronged approach to influence the nature and extent of exhaustion by promoting survival of antigen-responsive T cells (32) and controlling their localization and function (70).

## Chronic Infections and Coinhibitory Receptor Expression

Studies have shown that heterogeneity in coinhibitory receptor coexpression is apparent in different chronic disease settings and relevant for regulating T cell function to avert undue pathology. Here, we focus on T cell dysfunction and exhaustion that occurs during chronic infections, the concomitant upregulation of coinhibitory receptors, and the impact of checkpoint blockade on restoring antigen-specific T cell responses.

### Hepatitis B (HBV) Infection

Characterization of chronic hepatitis B peptide-specific T cells (surface antigen, core, envelope, and polymerase) based on expression of coinhibitory receptors and functionality (cytotoxicity, proliferation, and cytokine production) has been hampered by weak T cell responses observed in patients with chronic disease (76). In addition, the frequency of virus-specific T cells has been found to inversely correlate with low viremia (77). Nevertheless the study by Boni et al. almost a decade ago demonstrated that such HBV-specific peripheral CD8^+^ T cells from chronic patients expressed higher levels of PD-1 and could be restored by anti-PD-1 blockade (76, 78). Furthermore, in another study by the same group, expression of PD-1 was found to be higher on intrahepatic CD8^+^ T cells compared to their peripheral counterparts (79), indicating that cells in areas of virus tropism exhibit a more exhausted phenotype. Similar to the previous study, the function of exhausted intrahepatic CD8^+^ T cells could be restored by PD-1/PD-L1 blockade, demonstrated by increased production of IL-2. An interesting observation from both studies was the discrepancy in expression of IL-7R (CD127) between peripheral and intrahepatic CD8^+^ T cells. Intrahepatic HBV-specific CD8^+^ T cells expressed a lower percentage of CD127 compared to peripheral cells, which may be indicative of increased dysfunction. Expression of CD127 by effector CD8^+^ T cells is used to identify memory precursor cells and antigen-independent memory T cells that mediate protective immunity (80). In another study, high expression of CD127, which correlated with low expression of PD-1 was observed in HBV-specific CD8^+^ T cells from patients that resolved the infection (81). Other studies on HBV-specific CD8^+^ T cells as well other virus-specific CD8^+^ T cells have shown that downregulation of CD127 correlates with decreased functionality observed in chronic infections (82–85).

Differential expression of other coinhibitory receptors by HBV-specific T cells in chronic settings such as 2B4 (CD244), TIM-3, and its ligand, galectin-9 has also been described. HBV-specific CD8^+^ T cells compared to other virus-specific cells and total CD8^+^ T cells preferentially express TIM-3 (86). In a study by Raziorrouh et al., the occurrence and significance of high levels of CD244 on peripheral and liver HBV-specific CD8^+^ T cells from chronic patients compared to acutely infected patients was described. No correlation between CD244 expression and viral load was observed; however, HBV core and polymerase-specific CD8^+^ T cells expressed higher frequencies of CD244 (87). The authors also demonstrated that both peripheral and intrahepatic HBV-specific CD8^+^ T cells coexpress PD-1 and CD244. However, intrahepatic CD8^+^ T cells coexpressed higher levels of both receptors. Intriguingly, a significant increase in IFN-γ production upon antibody-blockade of CD244 or its ligand CD48 did not correlate with intracellular IFN-γ expression after antigen restimulation. Again this observation reflects the relationship between high expression of coinhibitory receptors and reduced functionality as mainly CD244^hi^ CD8^+^ T cells were “rescued” by antibody-blockade and subsequently produced higher levels of IFN-γ (87).

A similar study indicates the significance of TIM-3-galectin-9 binding in facilitating T cell exhaustion in hepatitis B infection (88). As observed in previously mentioned studies, HBV-specific CD8^+^ T cells from patients with chronic infection markedly expressed TIM-3, which correlated with reduced expression of IFN-γ, TNF-α, increased alanine aminotransferase (ALT) levels and induction of apoptosis (88). Mechanistically, results from this study suggest that liver Kupffer cells expressing galectin-9 present HBV antigens to TIM-3^+^ intra-hepatic T cells leading to cell dysfunction and exhaustion. Indeed, liver biopsies from chronic hepatitis B patients had a high intensity of galectin-9 and CD68 staining that correlated with serum galectin-9 levels and high ALT levels (>100 U/L), indicative of active infection. The authors also demonstrated the non-redundant roles of TIM-3 and PD-1 in mediating dysfunction of virus-specific T cells as coblockade of both coinhibitory receptors resulted in increased expression of IFN-γ and TNF-α. Interestingly CD8^+^ T cells from patients undergoing antiviral therapy also portrayed a “rescued” phenotype indicating the effectiveness of antibody coblockade in restoring T cell responses after viral suppression (88).

### Hepatitis C (HCV) Infection

Hepatitis C is characterized by viral persistence and dysfunctional virus specific CTLs. Accordingly, HCV is associated with upregulation of coinhibitory receptors on virus-specific CTLs. For instance, the coinhibitory receptors PD-1, CD160, 2B4, and KLRG1 are coexpressed by HCV-specific peripheral CD8^+^ T cells in about half of patients. Furthermore, coexpression of inhibitory receptors is controlled by ongoing viral antigen recognition and expression of CD127, which is indicative of T cell differentiation stage (89). Golden-Mason et al. first demonstrated expression of TIM-3 in CD4^+^ and CD8^+^ T cells in chronic HCV infection (90). The key finding from this study was the high frequency of PD-1^+^TIM-3^+^ HCV-specific CD8^+^ T cells in chronically infected patients and within the intrahepatic compartment. In a subsequent study, they showed that higher frequencies of PD-1^−^TIM-3^−^ HCV-specific CD8^+^ T cells are present in patients that acutely resolved the infection (91). In contrast PD-1^+^TIM-3^+^ double-positive CD8^+^ T cells were found primarily in the liver and had a central memory T cell phenotype, indicating extensive exhaustion in CD8^+^ T cells with enhanced proliferative potential (92, 93). These observations depict varying patterns of expression at different anatomical sites, which reflect different levels of CTL exhaustion. For instance HCV-specific CD8^+^ T cells in the liver of chronically infected HCV patients coexpress PD-1 and CTLA-4 compared to peripheral blood CD8^+^ T cells, reflecting more exhaustion at the site of viral replication (94). It has also been shown that splenic CD8^+^ T cells from patients with HCV-related cirrhosis are more exhausted compared to peripheral blood CD8^+^ T cells based on increased coexpression of PD-1 and TIM-3 and reduced percentage of IFN-γ producing cells (95). Splenomegaly, which occurs during chronic HCV following portal hypertension results in increased frequency of exhausted CD8^+^ T cells in the spleen (95). Sustained virological responses also influence the pattern of coinhibitory receptor coexpression (67). For instance, HCV-specific CD8^+^ intra-hepatic lymphocytes from patients with resolved infection coexpressed less PD-1, 2B4 and LAG-3 than cells from chronic HCV and CMV patients (67).

These studies on hepatitis B and C virus infections emphasize the significance of areas of virus tropism in influencing the level of exhaustion in responding CD8^+^ T cells. This has implications for further research on checkpoint blockade immunotherapy strategies as both preclinical murine and subsequent human clinical trials may have to focus mainly on phenotypic and functional characteristics of tissue resident and infiltrating effector T cells. Furthermore, the variability in the nature of inhibitory receptors coexpressed suggests that anatomical locations may also influence this occurrence (67).

### HIV

Chronic immune activation is a distinctive feature of HIV infection and a potential contributing factor to CTL exhaustion. Studies on HIV treatment-naive patients have shown that HIV-specific CD8^+^ T cells express high levels of PD-1 which directly correlates with increased plasma viral load and low CD4^+^ T cell count (96). Furthermore, similar to chronic HCV, HIV-specific CD8^+^ T cells expressing multiple coinhibitory receptors were found to be more dysfunctional compared to singly expressing cells as shown by PD-1 and CD160 coexpressing HIV-specific CD8^+^ T cells (97). PD-1^+^CD160^+^ CD8^+^ T cells are prominent during chronic HIV infection compared to PD-1^+^ and CD160^+^ single positive cells that predominated during acute HIV infection. By carrying out transcriptional profiling, PD-1^+^CD160^+^ double positive cells were found to upregulate genes involved in the inhibition of several survival pathways such as SUMO2, the small ubiquitin-like modifier, known to negatively regulate the activity of STATs (98). It has also been reported that there are significantly higher frequencies of HIV-1 Gag-specific CD8^+^ T cells coexpressing PD-1, CD160 and 2B4 compared to CMVpp65-specific CD8^+^ T cells (72). This study demonstrated that total CD8^+^ T cells from chronic HIV-infected patients display elevated levels of these markers and found this phenotype to correlate with high expression of the transcription factor EOMES (72). The EOMES^hi^ T-bet^dim^ profile displayed by total CD8^+^ T cells from chronically infected HIV patients has been shown to be indicative of irreversibly or terminally exhausted CD8^+^ T cells (73).

**Figure 3:**
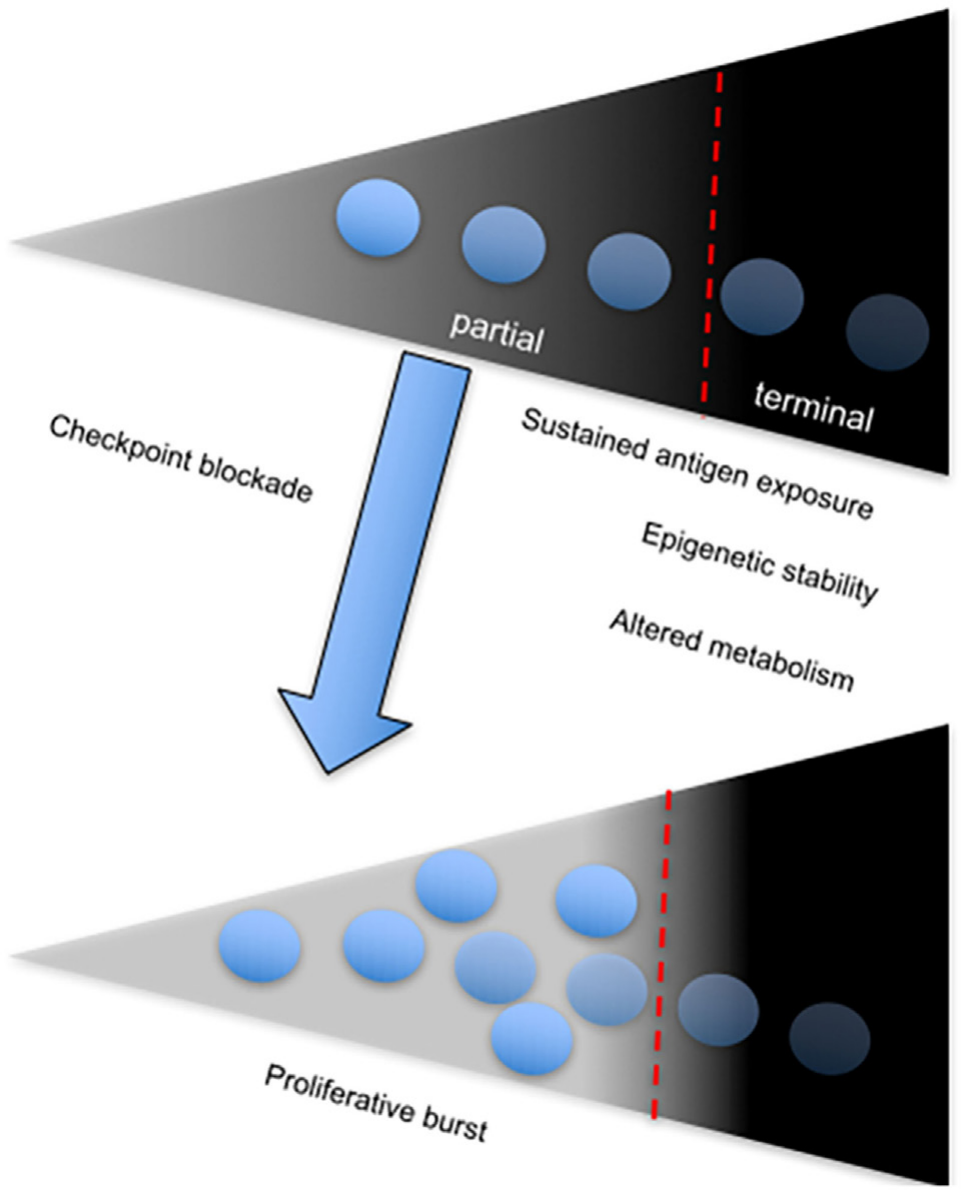
The onset and sustenance of T cell exhaustion is dependent on persistent antigenic stimulation. Diagram showing the T cell exhaustion gradient, which depends on sustained antigen exposure. The fate of chronically stimulated T cells is either partial or terminal exhaustion, which is associated with a stable chromatin landscape and altered metabolism. Partial-exhausted CD8^+^ T cells can be restored by checkpoint blockade, which leads to differentiation of the proliferative burst.

A direct correlation between failure to control viral replication and exhaustion in HIV infection with expression of the coinhibitory receptor, T cell immunoreceptor with Ig and ITIM domains (TIGIT) has been recently described (99). Among several observations, the authors showed that frequency of TIGIT^+^CD8^+^ T cells directly correlates with HIV disease progression depicted by comparing cohorts of acutely infected, combination antiretroviral therapy suppressed, elite controllers and non-controller HIV-infected individuals to healthy controls. Notably, they found that TIGIT^+^ CD8^+^ T cells coexpress PD-1 particularly within the non-controller group and this correlated with low CD4^+^ T cell counts and increased plasma viral load. Furthermore, TIGIT^+^PD-1^+^ double-positive CD8^+^ T cells displayed defective cytokine responses based on production of IFN-γ, IL-2, and TNF-α compared to single-positive PD-1^+^ or TIGIT^+^ CD8^+^ T cells (99). Coblockade of TIGIT and PD-L1 on PBMCs from chronically infected patients *in vitro* was effective at improving the proliferative ability of HIV-1 Gag-specific CD8^+^ T cells. However, *in vitro* coblockade only facilitated a significant increase in HIV-1 Gag-specific CD8^+^ T cells expressing IL-2, but not IFN-γ (99). These observations infer that coinhibitory receptors utilize various pathways to mediate immune control, depending on the nature of CTL responses and other extrinsic factors (Figure 3). The different effects of receptor blockade or coblockade on CTLs in the shape of cytokine responses, proliferative ability, cytotoxicity, and T cell subset preference also reflect the need for synergistic control of undue inflammation seen during chronic infections.

The significance of TIM-3 expression by CD8^+^ T cells from HIV-infected patients has been the subject of several studies. Jones et al. almost a decade ago investigated TIM-3 expression by peripheral blood T cells from treatment-naive, acutely infected and chronically infected HIV patients (100). They showed that CD8^+^ T cell intrinsic TIM-3 progressively increases with HIV infection stage and positively correlates with viral load. Furthermore, the percentage of TIM-3 expressed by CD8^+^ T cells inversely correlated with absolute CD4^+^ T cell counts, thus confirming an association between this receptor and chronic HIV infection. Other interesting findings from this study included the existence of a population of single-positive TIM-3^+^ CD8^+^ T cells distinct from TIM-3^+^PD-1^+^ double-positive CD8^+^ T cells in chronically infected patients (100). However, the authors did not investigate whether TIM-3^+^PD-1^+^ double-positive CD8^+^ T cells portrayed a more exhausted phenotype compared to TIM-3^+^ single-positive cells. Nonetheless, blockade of TIM-3-galectin-9 binding using soluble TIM-3 restored the ability of HIV-specific CD8^+^ T cells to proliferate and express IFN-γ.

We have also demonstrated upregulation of TIM-3 by HIV-specific CD8^+^ T cells, which positively correlates with susceptibility to Treg-mediated suppression (101). We have shown that HLA-B27 and HLA-B57-restricted CD8^+^ T cells, found predominantly in HIV-infected elite controllers, are protected from Treg-mediated suppression through TIM-3-Galectin-9 interaction. Such CD8^+^ T cells expressed lower levels of TIM-3 in response to stimulation by HIV epitopes compared to cells restricted by other non-protective HLA alleles such as HLA-A03, HLA-A24, HLA-A25, and HLA-A02. Importantly blockade of galectin-9 on Tregs by “masking” with glucose or small-interfering RNA knockdown averted the suppression of CD8^+^ T cells restricted by these non-protective HLA alleles. The mechanism underpinning resistance to suppression was found to be due to Granzyme B (GzmB)-mediated killing of Tregs by HLA-B27 and -57-restricted CD8^+^ T cells. This observation confirms that high expression of TIM-3 in progressive HIV infection compromises effector function of antigen-specific CD8^+^ T cells. Reduction in the production of IFN-γ by HLA-B27 and B57-restricted CD8^+^ T cells when cocultured with Tregs was attributed to the early time-point of the assay, before the onset of GzmB production (101). In agreement, increased levels of perforin and GzmB that correlated with low expression of TIM-3 in CD56^+^CD8^+^ T cells from HIV-positive elite controllers has been reported (102).

The negative effect of TIM-3 expression on CD8^+^ T cell effector function in HIV infection is represented by reduced cytotoxicity (103). Although TIM-3^+^ CD8^+^ T cells expressed more perforin than TIM-3^−^ cells, it was mainly in the granulated form, unable to mediate target cell killing. This correlated with reduced expression of the degranulation marker CD107a. Blockade of TIM-3 using antibodies restored degranulation and perforin release in addition to production of GzmB by HIV-specific CD8^+^ T cells (103). These studies suggest that TIM-3 plays a key role in orchestrating the regulation of CD8^+^ T cell responses in chronic HIV. Nevertheless, it will be interesting to clarify whether coexpression of other inhibitory receptors complement the activity of TIM-3 in chronic HIV infection.

### Coinfections

CD8^+^ T cells elicited in response to coinfections exhibit a more exhausted phenotype compared to cells from monoinfections (104, 105). A study by Vali et al. compared PD-1 and TIM-3 coexpression on HCV-specific CD8^+^ T cells during HIV/HCV coinfection (106). They found that HCV-specific CD8^+^ T cells from coinfected patients coexpressed higher frequencies of PD-1 and TIM-3 than HCV-specific cells from monoinfection and this positively correlated with progression to liver disease. Furthermore, coexpression of PD-1 and TIM-3 was significantly higher on HCV-specific CD8^+^ T cells compared to HIV-specific CD8^+^ T cells. Antibody blockade of PD-1 or TIM-3 expressed by PBMCs restored both HCV- and HIV-specific CD8^+^ T cell proliferation and cytokine production (106). A similar study showed that coinfected patients with HCV/HIV displayed lower frequencies of effector memory CD4^+^ and CD8^+^ T cells compared to HCV monoinfected patients and these cells expressed higher percentages of PD-1 and TIM-3 (107). In addition, effector memory CD8^+^ T cells that expressed PD-1 or TIM-3 were less cytotoxic based on CD107a degranulation and produced less IFN-γ. These observations demonstrate that intricate as well as complex differences in chronic disease phenotypes and the nature of corresponding immune responses contribute to different levels of CTL exhaustion marked by a varied inhibitory receptor profile.

## Cancer

Heterogeneity in coinhibitory receptor expression by exhausted CD8^+^ T cells seen in various types and stages of chronic infections imply that tumor-associated exhausted CD8^+^ T cells may exhibit a different coinhibitory receptor profile. Furthermore, coinhibitory receptor expression may depend on cancer type and stage. Various tumors express PD-L1, which binds to PD-1 on T cells leading to immunosuppression and tumor evasion (108, 109). Indeed, several studies have shown that PD-1 expression by tumor-infiltrating T cells is the main indicator of functional impairment and heterogeneity in responses to immune checkpoint blockade (110–112). It is unclear whether the occurrence and frequency of neoantigens impact already exhausted tumor-infiltrating T cells (TILs), although neo-antigen-specific CD8^+^ T cells have been identified by PD-1 expression (113). However, resistance to cancer immunotherapies including PD-1 blockade and concomitant upregulation of other inhibitory receptors has elucidated the role and significance of immune checkpoint coinhibition on tumor control (114).

Similar to exhausted CD8^+^ T cells associated with chronic infections, tumor infiltrating lymphocytes that coexpress multiple coinhibitory receptors display a more exhausted phenotype compared to single-positive and double-negative cells represented by impaired T cell function (e.g., cytokine production and cytotoxicity) (63, 115–119).

### Metastatic Melanoma

Immune responses to metastatic melanoma have been well characterized, forming the basis for adoptive cell therapy, development of dendritic cell vaccines, and immune checkpoint blockade (18, 40, 120–122). In a study by Steven Rosenberg’s group almost a decade ago, tumor-infilitrating T cells including MART-1/Melan-A-specific CD8^+^ T cells present in metastatic melanoma lesions were found to predominantly express PD-1, compared to T cells in normal tissues and peripheral blood (110). A recent study by Kleffel et al. has shown that melanoma-intrinsic PD-1 promotes tumor growth (123). Immunofluorescent staining of melanoma patient biopsies showed that PD-1 and MART-1 colocalize in tumor lesions (123). PD-1^+^ CD8^+^ TILs express lower percentages of IL-2 and IFN-γ than PD-1-negative cells, they also express CTLA-4; indicating that these cells are functionally impaired (110). In another study, the percentage of PD-1^+^ peripheral blood NY-ESO-1-specific CD8^+^ T cells from stage IV melanoma patients was approximately five- to sixfold higher than MART-1-specific CD8^+^ T cells (124). Although expression of PD-1 by NY-ESO-1-specific cells did not compromise IFN-γ and TNF-α production in response to non-specific stimulation, stimulation of NY-ESO-1-specific CD8^+^ T cells with cognate antigen in the presence of α-PD-L1 blocking antibody significantly increased proliferation and the percentage of IFN-γ^+^ and TNF-α^+^ cells. In this study, PD-L1 antibody blockade had no effect on cytokine expression by MART-1-specific CD8^+^ T cells, indicating that PD-1-PD-L1 signaling negatively regulated NY-ESO-1-specific CD8^+^ T cells (124). These early studies show that melanoma antigen-specific CD8^+^ T cells present in the tumor microenvironment exhibit more “exhaustion” characteristics compared to peripheral blood cells. Furthermore, the level of exhaustion may depend on antigen-specificity, PD-1 expression and regulation by PD-1-PD-L1-signaling (110, 118, 125).

In a gene-profiling study of tumor-specific CD8^+^ T cells from melanoma patients, MART-1-specific CD8^+^ T cells obtained from metastases expressed a large variety of T cell exhaustion genes (117). CD8^+^ T cells isolated from tumor-infiltrated lymph nodes (TILNs) were enriched in CTLA-4, CD160, 2B4, LAG-3, PTGER4, and PD-1 compared to their peripheral blood-derived counterparts. Furthermore, tetramer-positive TILN CD8^+^ and peripheral blood CD8^+^ T cells from melanoma patients in this study expressed similar levels of 2B4, PD-1, TIM-3, and CD160. Nevertheless, antigen-specific TILN CD8^+^ T cells had significantly higher frequencies of LAG-3 and CTLA-4 compared to peripheral blood CD8^+^ T cells (117). There were also differences in the coexpression pattern and frequency of coinhibitory receptors obtained from the TILN and peripheral blood. Analyses of MART-1-specific CD8^+^ T cells showed coexpression of three to four inhibitory receptors by TILN CD8^+^ T cells in comparison to one or two expressed by peripheral blood CD8^+^ T cells (117). In addition TILN CD8^+^ T cells produced significantly less IFN-γ compared to peripheral blood CD8^+^ T cells (117). Peripheral blood MART-1-specific CD8^+^ T cells in this study exhibited a late effector T cell phenotype based on high expression of perforin and GzmB (126, 127). In addition, the main coinhibitory receptor expressed by these cells was KLRG-1, a marker of cell senescence. These observations indicate that antigen-specific CD8^+^ T cells present in the periphery exhibit a partially exhausted phenotype in contrast to TILNs, which are more functionally impaired and coexpress multiple inhibitory receptors (117). These findings are similar to the “more exhausted” phenotype seen in antigen-specific CD8^+^ T cells from the liver and spleen compared to the peripheral blood of HCV patients (90, 91). It will be interesting to determine whether such dichotomy is evident in other chronic infections such as HIV and HBV.

A more comprehensive study by the same group has shown that the composition and numbers of coinhibitory receptors expressed by MART-1-specific CD8^+^ T cells depend on the microenvironment, vaccination, and differentiation state (116). The expression of eight coinhibitory receptors by MART-1-specific CD8^+^ T cells: BTLA, TIM-3, LAG-3, KLRG-1, 2B4, CD160, PD-1, and CTLA-4 was investigated. By comparing the phenotype of total TILN CD8^+^ T cells with peripheral blood CD8^+^ T cells they found that LAG-3, CTLA-4, and TIM-3 were upregulated in the former. In addition, they showed that MART-1-specific TILN CD8^+^ T cells expressed more TIM-3, LAG-3, and CTLA-4 than non-specific TILN CD8^+^ T cells. However, expression of PD-1, CD160, 2B4, and BTLA was comparable between the two groups (116). Although these observations were from MART-1/Melan-A-specific CD8^+^ T cells, they correlate with other findings, which show that exhausted T cells in the periphery and microenvironment broadly express PD-1 whilst coexpression with other inhibitory receptors is dependent on the type and stage of tumor or chronic infection (111).

The presence of partially functional and exhausted CD8^+^ T cells in melanoma lesions is in contrast to observations in chronic infections where cells at sites of virus tropism display a more exhausted phenotype compared to their peripheral counterparts. It is unclear whether heterogeneity in the functional ability of TIL CD8^+^ T cells depends on the frequency of antigen specific T cells present in the tumor microenvironment. The ability of some tumors to promote T cell exhaustion due to upregulation of PD-L1 may also contribute to this phenomenon (123).

The potential of less studied inhibitory receptors such as TIGIT in regulating melanoma-associated CD8^+^ T cell responses has been outlined in two recent studies (63, 128). Both studies showed that TIGIT can only regulate the function of melanoma-specific CD8^+^ T cells when it is coexpressed with PD-1. Furthermore, expression of the TIGIT ligand, CD155 by melanoma cells or APCs and downregulation of CD226, which competes with TIGIT for binding to CD155, are also required for TIGIT-mediated regulation of antimelanoma CD8^+^ T cells responses (63, 128). This also implies that coexpression of high levels of PD-1 with other inhibitory receptors is a prerequisite for regulation of antimelanoma CD8^+^ T cell responses in tumors.

It is likely that expression of PD-L1 by melanomas and other tumors evolved to circumvent the activity of tumor infiltrating T cells, which predominantly express PD-1. However, it is still unclear whether variations in the coinhibitory receptor composition reflect severity, anatomical location, or antigen specificity. Studies that investigated coinhibitory receptor expression by NY-ESO-1-specific CD8^+^ T cells from advanced melanoma patients have shown that coexpression of PD-1 with TIM-3, BTLA and TIGIT indicate T cell dysfunction and exhaustion (20, 63, 118). However, BTLA expression is independent of progressive stimulation by cognate antigen and subsequent CD8^+^ T cell dysfunction as BTLA^+^ (BTLA^+^PD-1^+^TIM-3^−^) CD8^+^ T cells did not lose the ability to produce IL-2 (20). This observation suggests that (co)-expression of coinhibitory receptors such as PD-1 and Tim-3, are more indicative of CD8^+^ T cell exhaustion compared to others. It also shows that “more exhausted” tumor-specific CD8^+^ T cells coexpress higher numbers of inhibitory receptors (16). Interestingly blockade of BTLA had an “additive” effect on PD-1 and TIM-3 coblockade portrayed by increased proliferation and IL-2 production by NY-ESO-specific CD8^+^ T cells (20). The presence of a population of BTLA^+^PD-1^+^TIM-3^−^ partially exhausted CD8^+^ T cells has implications for therapeutic interventions. BTLA has been shown to be expressed by naive MART-1/Melan-A CD8^+^ T cells and is gradually downregulated in response to vaccination with CpG-ODN in melanoma patients (116). Yet, persistent expression of BTLA has been observed in untreated melanoma patients and patients vaccinated without CpG (129). BTLA^+^PD-1^+^TIM-3^−^ CD8^+^ T cells expressed less PD-1 compared to their triple-positive counterparts and were hence less dysfunctional (20).

It is apparent that coexpression of PD-1 with multiple (two or three) coinhibitory receptors by CD8^+^ TILs correlates with the level of T cell exhaustion. Partially or terminally exhausted CD8^+^ TILs are designated based on functionality and restoration upon antibody-blockade treatment (Figure 2). However, it is likely that PD-1 expression is required due to specific blockade and expression of PD-L1 by tumors and tumor-associated myeloid cells.

The coexpression of PD-1 and CTLA-4 (PD-1^hi^CTLA-4^hi^) by TIL CD8^+^ cells from metastatic melanoma tumors has been shown to correlate with a positive response to anti-PD-1 monotherapy (112). PD-1^hi^CTLA-4^hi^ CD8^+^ TILs were found to be partially exhausted based on their ability the produce IFN-γ but not TNF-α and IL-2 (112). Also, in a mouse proof-of-concept study, therapeutic responses to PD-1 blockade were assessed based on levels of PD-1 expression (PD-1^hi^ and PD-1^lo^) by T cells and both myeloid and T cell PD-L1 expression (PD-L1^hi^) in the tumor microenvironment (130). High frequencies of PD-1^lo^ partially exhausted T cells were shown to directly correlate with a positive response to PD-1 blockade in sensitive (PD-L1^lo^) and resistant (PD-L1^hi^) tumors. In contrast PD-1^hi^ T cells present in resistant tumors were not rescued by PD-1 blockade (130). These studies suggest that the efficacy of PD-L1-dependent anti-PD-1 therapy has to be confirmed by demonstrating if engagement of PD-1 with PD-L1 occurred. A recent study has demonstrated that levels of Bim (BCL-2-interacting mediator of cell death) expressed by tumor-reactive CD8^+^ T cells from melanoma patients indicates engagement of PD-1 with PD-L1 (131). The authors also showed that expression of Bim can predict clinical benefit in melanoma patients treat with pembrolizumab (anti-PD-1) (131).

The presence of distinct populations of CD8^+^ T cells in the tumor microenvironment based on cytokine production, cytotoxicity, proliferative capacity and coinhibitory receptor coexpression indicates that tumors can drive differentiation of heterogeneous populations of exhausted T cells and chronically activated effector T cells (132). These chronically activated effector CD8^+^ T cells maintain expression of activation markers such as CD69 and Ki67, express PD-1 and BTLA, are weakly cytotoxic and able to undergo proliferation thereby facilitating tumor control (133). Alternatively TILNs that express PD-1 and portray moderate functionality compared to their counterparts that coexpress PD-1 and multiple inhibitory receptors may be reversible exhausted CD8^+^ T cells, reminiscent of cells observed during CMV infection characterized by a balanced expression pattern of the transcription factors T-bet and Eomes (72). Expression of T-bet has been shown to promote IFN-γ production and cytotoxicity by TILN cells (134).

The above-mentioned studies and others reveal complexities in the nature of “exhausted” CD4^+^ and CD8^+^ T cells during tumor development and progression (125). It is apparent that differentiation and infiltration of tumor antigen-associated cells to affected sites and expression of coinhibitory receptor ligands such as PD-L1 by tumors contribute to the heterogeneity in effector function and subsequent tumor control and patient survival. Therefore, we may conclude that the coinhibitory profile and function of exhausted T cells seen in the periphery and within tumors of cancer patients is dependent on multiple factors ranging from tissue type, disease stage, and other extrinsic factors (Figure 3). Nevertheless, like exhausted T cells from chronic infections, coexpression of multiple inhibitory receptors, particularly PD-1, serves as an indicator of the severity of the disease and specificity of responding T cells. With the increasing use of genomic approaches such as RNA sequencing to identify pathways, susceptibility genes and biomarkers of disease, such techniques can be adopted to characterize the exhaustion phenotype at several levels. In a recent study by Tirosh et al. results from single cell RNA sequencing of CD8^+^ T cells from melanoma tumors identified a core exhaustion signature independent of T cell activation, comprising of genes encoding inhibitory receptors (TIM-3, PD-1, LAG-3, CTLA-4) and NFATC1 (49).

## Immune Checkpoint Blockade

**Figure 4:**
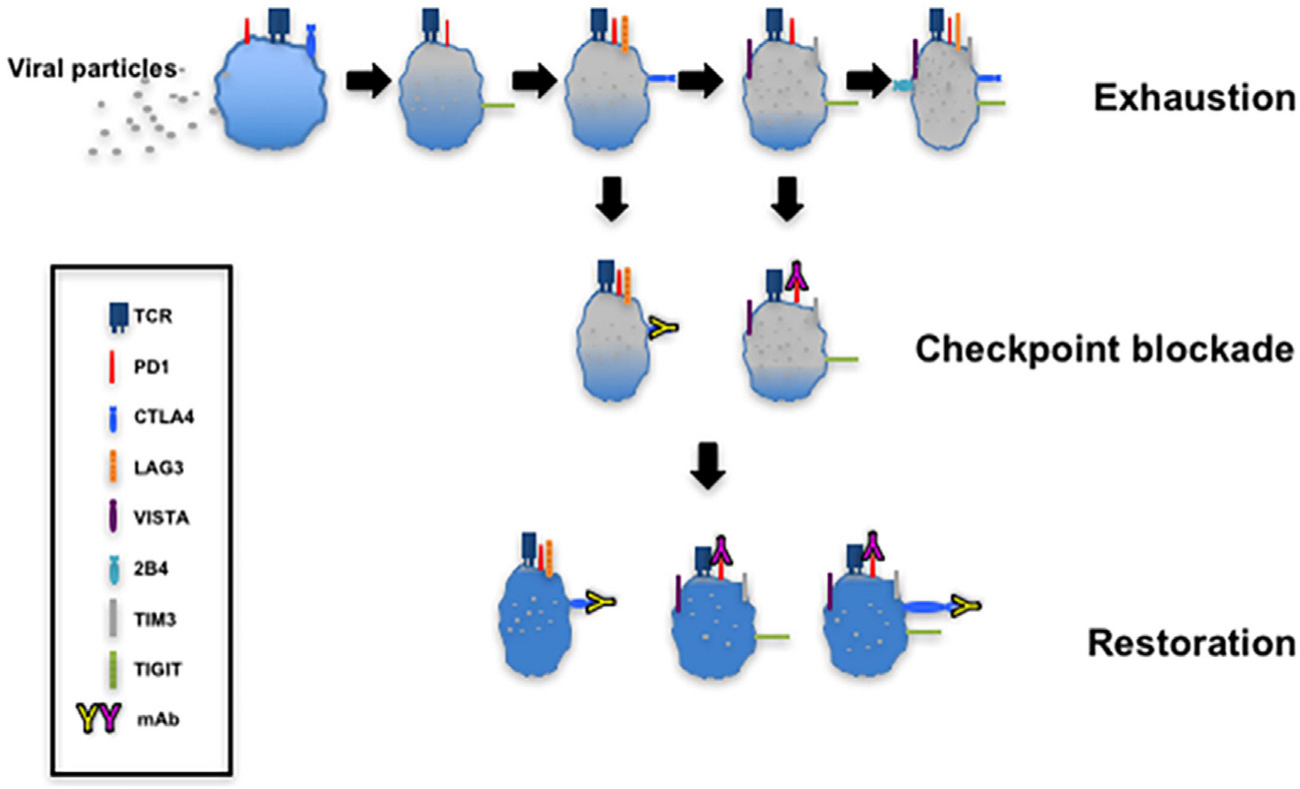
T cell exhaustion and restoration. This model depicting progressive exhaustion of CD8^+^ T cells during viral infection directly correlates with coexpression of immune checkpoints. Checkpoint blockade implemented by administration of monoclonal antibodies such as α-programmed cell death 1 (α-PD-1) and α-cytotoxic T lymphocyte antigen-4 (α-CTLA-4) restores the function of exhausted T cells.

The targeting of upregulated coinhibitory receptors and their ligands in response to microbial invasion or tumor development forms the basis of immune checkpoint blockade that restores CTL function (Figure 4). There are several clinical trials registered or completed that target inhibitory receptors as discussed below (https://www.clinicaltrials.gov).

### Cancer

In 2011, the United States Food and Drug Administration (FDA) approved the use of ipilimumab, a monoclonal antibody targeted at CTLA-4, for the treatment of melanoma (135). Clinical trials on the use of other monoclonal antibody-based drugs that target CTLA-4 (tremelimumab) (136–139), PD-1 (pembrolizumab, nivolumab, and pidilizumab) (140–142), and PD-L1 (BMS-986559, MPDL3280A, and MEDI4736) (143–145) as treatment strategies for melanoma, renal cell carcinoma, and non-small cell lung cancer have either been completed or ongoing. The FDA approved treatment of patients with metastatic melanoma and disease progression following ipilimumab treatment and with the BRAF V600 mutation with pembrolizumab in 2014 (146). Importantly, the blockade of CTLA-4 and PD-1/PD-L1 is being extended to the treatment of other cancers (147, 148) and combination therapies, which utilize checkpoint inhibitors with other immunotherapy approaches, are being explored [reviewed in Ref. (18)]. The use of nivolumab in combination with ipilimumab for the treatment of advanced melanoma patients with or without BRAF V600 wild-type metastatic melanoma has also been approved (149, 150).

The use of monoclonal antibodies that target TIM-3 and LAG-3 as cancer therapeutic agents has also been investigated. Plans to start a phase I-Ib/II trial to investigate the safety and efficacy of the anti-TIM-3 antibody MBG453 as a single agent or in combination with the anti-PD-1 antibody PDR001 in adult patients with solid tumors has been scheduled (151). Similarly, a phase I/II study using LAG525, a monoclonal antibody that targets LAG-3, to mediate checkpoint blockade alone or in combination with PDR001 in patients with advanced malignancies has been initiated by Novartis (152).

The use of LAG-3 as an adjuvant in cancer vaccines and chemotherapy has also been documented (153). Two phase I clinical trials that investigated the use of IMP321 (a recombinant soluble human LAG-3-Ig fusion protein) to treat stage IV metastatic renal cell carcinoma and metastatic breast carcinoma with Paclitaxel were completed in 2008 and 2010, respectively (154, 155). The LAG-3-Ig fusion protein is a non-TLR agonist that binds to MHCII with stronger affinity than CD4, eliciting DC stimulation and subsequent activation of CD8^+^ T cells (156). In a recent phase I/II clinical study a combination of LAG-3 Ig and peptides from five tumor-associated antigens was able to induce antigen-specific CD4 and CD8^+^ T cell responses in metastatic melanoma patients (157). Plans to study the effectiveness of IMP32I as an adjuvant to anti-PD-1 (Pembrolizumab) therapy in metastatic melanoma are underway (158).

### Immune Checkpoint Blockade and Chronic Infections

Checkpoint blockade as a means of restoring CTL activity during chronic infections has been proposed although there is a higher risk of triggering exacerbated responses by peripheral and tissue lymphocytes. Several studies have demonstrated that blockade of PD-1 expressed by exhausted virus-specific CD8^+^ T cells alone, or in combination with other inhibitory receptors *in vitro* can improve their effector function (69, 76, 99). Nevertheless preclinical *in vivo* animal studies to evaluate the efficacy and the mechanisms underlying checkpoint blockade are essential steps in the development of this strategy [reviewed in Ref. (159)]. Antibody treatment of SIV-infected macaques and HIV-infected humanized mice have provided valuable insight to the effectiveness of checkpoint blockade in restoring virus-specific CD8^+^ T cell responses(160–163). Early studies based on blockade of PD-1 and CTLA-4 in chronic SIV-infected macaques showed improved SIV-specific immunity in the blood and gut as well as decreased viral loads in antibody-treated animals (160, 161). A recent study using bone marrow–liver–thymus (BLT) humanized mice chronically infected with HIV-1, demonstrated significant reduction in viral load in response to PD-1 blockade (45-fold compared to untreated mice) after 4 weeks of treatment (162). Interestingly, PD-1-blockade resulted in increase of CD8^+^ T cell numbers but not viral-specific humoral responses. Results from this study demonstrate the specificity of PD-1 blockade, which targeted PD-1^hi^ CD8^+^ T cells and not cells that expressed other inhibitory receptors such as 2B4, CD160 and LAG-3 (162). Fuller et al. investigated T cell responses after anti-PD-1 treatment of chimpanzees infected with chronic HCV (164). PD-1 blockade led to the reduction in HCV viremia without hepatocellular injury. They showed that control of viral replication is dependent on the restoration of CD4^+^ and CD8^+^ intrahepatic T cells specific for multiple HCV proteins (164).

Following confirmation of the effectiveness in animal models, the main focus of current clinical studies is on the safety and efficacy of monoclonal antibodies for the treatment of human virus infections. This has been demonstrated in a recent study in which HIV-1-infected adults on cART treatment and detectable viral RNA were treated with the anti-PD-L1 antibody BMS-936559 (165). In two out of six study participants, HIV-1-specific responses increased in response to infusion (0.3 mg/kg) based on expression of IFN-γ, CD107a and TNF. Importantly, patients did not exhibit any treatment-related grade 3 or higher immune-related adverse events (IRAEs) (165, 166). In a proof-of-concept study by Gardiner et al. a single dose of a monoclonal antibody to PD-1 (BMS-936558/MDX-1106) was administered to patients with chronic HCV infection (167). They observed HCV RNA reductions in 3 out of 20 patients treated with a high dose of antibody (10 mg/ml). They also reported that two patients also treated with high dose of BMS-936558, one of which was a null-responder to IFN-α treatment, achieved HCV RNA quantitation below the limit of detection (167). Although these observations need to be validated using a larger sample size, they pave the way for the application of checkpoint blockade in other chronic infections.

A number of clinical trials are investigating the side effects, dosage, and efficacy of PD-1 and CTLA-4 monoclonal antibodies for treating patients with HIV- and hepatitis-associated cancers [reviewed in Ref. (168, 169), (170, 171)]. In a study by Sangro et al., the antitumor and antiviral effects of tremelimumab (anti CTLA-4 monoclonal antibody) was tested in patients with hepatocellular carcinoma patients (HCC) with or without associated HCV infection (172). Tremelimumab treatment yielded a good safety profile with limited adverse effects, a partial response rate of 17.6%, and a moderate disease control rate (76.4%). A significant reduction in viral load associated with increased anti-HCV immune responses were also observed, paving the way for further investigation of such immunotherapy strategies (172). The outcome of the CheckMate 040 study, in which nivolumab (anti-PD-1 antibody) was administered to patients with advanced HCC with or without chronic viral hepatitis has recently been published (173). These studies demonstrate the potential of PD-1 and CTLA-4 monoclonal antibodies to elicit durable objective responses with manageable safety profiles when used for treatment of HCC (172, 173). PD-1 blockade treatment of two advanced melanoma patients infected and coinfected with HIV and HCV, respectively, has also been described (43). Treatment with pembrolizumab in conjunction with ART, facilitated a stable HCV/HIV viral load in patient 2; however, treatment was halted as a result of disease progression. Treatment in patient 1, on the other hand, resulted in stable HCV viral loads followed by a complete decline in response to additional anti-HCV treatment (sofosbuvir) after nine cycles of pembrolizumab (43). A phase I trial to study the effectiveness of nivolumab administered in combination with ipiliimumab (anti-CTLA-4 antibody) for treating patients with HIV-associated classical Hodgkin’s lymphoma has been set up by the National Cancer Institute (174). These progresses in determining the safety and efficacy of anti-PD-1 and CTLA-4 monoclonal antibodies in the treatment of human chronic virus infections pave the way for checkpoint blockade strategies utilizing other monoclonal antibodies such as TIM-3 and LAG-3.

### Global T Cell Function in Response to Checkpoint Blockade: Storm after the Calm?

Based on the heterogeneity of inhibitory receptor expression by T cells the uncoupling of immune regulation in response to checkpoint blockade may have more far-reaching effects than the restoration of CD8^+^ T cell effector responses. In this regard, checkpoint blockade strategies should take into account the potential elicitation of other T cell subsets and their ability to promote autoimmunity or local immunopathological responses. For instance, the constitutive expression of CTLA-4 by FOXP3 + Tregs (175) has implications for CTLA-4-based checkpoint blockade. CTLA-4-deficient mice develop lymphoproliferative disease associated with autoimmune spectra such as pancreatitis and myocarditis (176). Also in a recent study by Klocke et al., the effect of congenital CTLA-4 deficiency compared to conditional deletion in adult mice was compared (177). Deletion of CTLA-4 in adult mice resulted in varying tissue pathologies; however, this was not fatal. Interestingly, CTLA-4 deletion promoted expansion of defective FOXP3 + Tregs that were unable to control the development of peptide-induced experimental autoimmune encephalomyelitis in affected mice (177). Such findings indicate that CTLA-4-based checkpoint blockade may preferentially target Tregs and hence induce gross autoimmune responses. Indeed a number of immune-related adverse events in response CTLA-4 blockade have been reported (see Immune-Related Adverse Events).

The compensatory upregulation and further T cell suppression by other inhibitory receptors coexpressed with blockade antibody targets by CD8^+^ T cells has been observed (74, 75). This may be dependent on the level of coexpression and the abundance of coexpressing T cells. In the study by Seung et al., PD-1 blockade in HIV-infected BLT mice did not affect coexpressing 2B4, CD160, and LAG-3 (162). However, the alternative upregulation of TIM-3 by PD-1-bound CD8^+^ T cells in response to anti-PD-1 treatment in a mouse lung adenocarcinoma model and in lung cancer patients (75) implies this occurrence can compromise the effectiveness of checkpoint blockade. Furthermore, in a recent study by Gao et al., the authors reported increased protein expression of VISTA, PD-1, and PD-L1 protein in tumors of ipililmumab-treated metastatic prostate cancer patients (74). Prostate cancer has been shown to be poorly responsive to checkpoint blockade monotherapy (178), which could be attributed to the compensatory upregulation other inhibitory receptor pathways upon ipilimumab treatment. They carried out comprehensive analyses of tumor tissues and blood from treated patients and detected increased PD-L1 and VISTA expression. In addition they observed increased expression of these markers by CD4^+^, CD8^+^T cells, and CD68^+^ macrophages in treated patients (74). Comparative analyses of posttreatment prostate and melanoma tumors showed significantly greater proportions of VISTA expressed by CD68^+^ macrophages from prostate tumors (74).

These observations indicate factors that should be taken into consideration when determining checkpoint blockade therapies. The enhancement of cell infiltration into tissues in response to anti-CTLA-4 treatment is possibly triggered by reduced Treg expansion and activity. The potential development of autoimmune responses suggests that measures should be adopted to determine safety levels of CTLA-4 administration and longitudinal characterization of infiltrating immune cells to determine phenotype, activation status and inhibitory receptor profile. For cancer treatment, the tumor responses to monotherapy as well as the nature of immune responses elicited must be taken into account. The ability of checkpoint blockade to trigger expansion of T cells and monocytes, which can be mediators of adaptive resistance (75) and further immune suppression must be addressed. The onset of checkpoint blockade studies targeting novel inhibitory receptors such as TIGIT and VISTA will help identify how such receptors influence immune responses (38).

### Combination Therapy

Monotherapies involving the use of anti-CTLA-4 and PD-1 monoclonal antibodies have proven efficacy in the treatment of melanoma and non-immunogenic tumors such as non-small lung cell cancer and renal cell carcinoma. Nevertheless, objective responses and progression-free survival have been shown to depend on factors that compromise patient responses to therapy such as PD-L1 tumor expression and mutations. Lack of redundancy and synergistic responses observed due to coinhibitory receptor expression has been harnessed as a strategy to improve antigen-specific CD8^+^ T cell responses in tumors and chronic infections (18, 179, 180). Early studies by Wolchok et al. investigated the effectiveness of anti-CTLA-4 and PD-1 combination therapy compared with monotherapy or sequential treatment using anti-CTLA-4 blockade followed by anti-PD-1 checkpoint blockade in unresectable melanoma patients. Data obtained from this study suggested higher objective response rates as patients showed more durable responses to combined treatment with nivolumab and iplimumab compared to other treatments tested (181). Similarly, patients with PD-L1-negative tumors treated with anti-PD-1 and CTLA-4 coblockade had a progression-free survival of 11.2 months compared to PD-1 and CTLA-4 monotherapy patients that had a progression-free survival of 5.3 and 2.8 months, respectively (40). Adverse effects as a result of combination blockade have also been reported [reviewed in Ref. (182)]. Nevertheless, grade 3 and 4 irAEs, which occurred in a higher percentage of combined treatment patients, were manageable and treated using immunomodulatory agents and secondary immunosuppressive agents (e.g., the TNF inhibitor, infliximab). These results demonstrated the safety and effectiveness of nivolumab and ipilimumab combination therapy (approved by the FDA on October 1, 2015, for the treatment of advanced melanoma) and pave the way for other coblockade strategies using other immune checkpoint inhibitors. Plans to start a phase I-Ib/II trial to investigate the safety and efficacy of the anti-TIM-3 antibody MBG453 in combination with the anti-PD-1 antibody PDR001 or as a single agent in adult patients with advanced solid tumors has been scheduled (183).

### Immune-Related Adverse Events

The expression of coinhibitory receptors/immune checkpoints and their ligands by T cells and antigen-presenting cells evolved as a “molecular brake” system to mediate immune tolerance and contain immunopathology in response to inflammation and microbial invasion. Consequently, there is the risk of eliciting undue immune activation or autoimmune responses from the use of checkpoint blockade as an immunotherapy strategy. Indeed the efficacy and safety of checkpoint inhibitors has been of concern and several toxic episodes termed irAEs have been reported (166, 184–189). The National Cancer Institute in 2010 created a grading system for reporting adverse events ranging from grade 1 (mild) to grade 5 (death) and based on system organ class (190). Adverse events usually manifest in the form of skin inflammation (191–195), gastrointestinal disorders (196–200), hepatic malfunction (191, 196), respiratory complications (186, 197), and endocrine disorders (191, 198). Rare manifestations such as sicca syndrome and arthritis (199), ocular inflammation (197, 200, 201), nephritis (202–204), heart disease (197, 205), and meningitis (206) also occur. Various case studies and clinical trials have demonstrated that there is less toxicity and prolonged progression-free survival from the use of PD-1 checkpoint blockade compared to CTLA-4, in advanced melanoma, for instance (187, 207). Also, it has been shown that in a randomized, double-blind phase III study that patients with unresectable stage III or IV melanoma treated with nivolumab have a higher progression-free survival rate compared to ipilimumab-treated patients (40). The ability of checkpoint blockade, particularly anti-CTLA-4-based treatments to perturb the Treg-effector T cell balance may be an underlying cause of IRAE development. CTLA-4 depletion has been shown to promote lymphoproliferative disease and gross immunopathology in deficient mice (176). Also studies have shown that ipilimumab administration can facilitate killing of Tregs by antibody-dependent cell-mediated cytotoxicity (208). Genomic approaches have been used to identify biomarkers, which may indicate favorable responses as well as toxicities associated with the use of checkpoint blockade inhibitors in cancer immunotherapy. Molecules such as the proliferation marker Ki67, inducible T cell costimulator, EOMES and granzymes by CD4^+^ and CD8^+^ T cells isolated from tumors and the periphery are differentially regulated in response to anti-PD-1 or CTLA-4 monotherapy and combination therapy and have been shown to be associated with the development of irAEs (209, 210).

Several strategies for managing irAEs in response to ipilmumab and other checkpoint inhibitors have been proposed, which include treatment interruption, risk assessment, patient stratification and the use of immunosuppressive drugs such as corticosteroids and TNF-blocking antibodies (211–215).

### Small but Mighty-Small Molecules As Immune Checkpoint Inhibitors

Targeting intracellular and extracellular mechanisms that occur during tumor development using small molecule inhibitors alone or in conjunction with other therapies has been proposed as a cancer immunotherapy strategy [reviewed in Ref. (216)]. Small molecule inhibitors that target pathway components such as BRAF^V600E^ (signal transduction) have been approved for metastatic melanoma treatment (217, 218) and clinical trials testing mediators such as IDO, ARG1, ARG2 (amino acid catabolism) are underway (https://www.clinicaltrials.org). In addition a study to evaluate the safety and activity of CA-170, a small molecule that directly targets PD-L1/L2 and VISTA, for treating patients with advanced solid tumors and lymphomas has been recently set up (219).

The recent discovery of the crystal structure of the human PD-1–PD-L1 binding complex (220) in addition to previous reports on the structure of mouse PD-1 and human PD-L1 and PD-L2 binding complexes (221) has increased the possibility of using small molecules as immune checkpoint inhibitors. Small molecule inhibitors have huge potential to overcome the limitations associated with antibody-based immune checkpoint blockade, particularly long half-life *in vivo* and possible development of iRAEs, global patient accessibility, production and logistic costs [reviewed in Ref. (222–225)].

A number of sulfonamide derivatives that modulate the activity of PD-1 in PD-1 transgenic cells compared to knockout cells based on an IFN-γ release assay have been discovered by researchers at Harvard University (WIPO, patent number WO2011082400). Compounds (BMS-230, -242, -8, and -37) with tri-aromatic structures and a methanol scaffold have been described by Bristol-Myers Squibb (BMS) and shown to block PD-1-PD-L1 protein–protein interaction in a homogeneous time-resolved fluorescence binding assay (HTRF) (WIPO, patent number, WO2015034820). In addition, a recent study has characterized the interaction of BMS compounds with PD-1 and PD-L1 using a nuclear magnetic resonance (NMR) (226). An NMR approach, which enables small organic molecules that bind to proximal subsites of protein to be identified, was used. The authors showed that BMS compounds bind to PD-L1 but not PD-1 and two are able to mediate dissociation of PD-1-PD-L1 interaction. Furthermore, they found that BMS compounds induce dimerization of human PD-1 in solution (226). A key finding from this study was the identification of “hotspots” and residues at the surface of PD-L1 that can serve as potential targets for small molecule inhibitors.

These observations show the potential of small molecule inhibitors to become the next generation of cancer therapeutics with great clinical and economic implications. This has prompted the design and development of novel small molecule inhibitors that target PD-1 and PD-L1 outside the chemical space of BMS molecules using computational modeling. Expected challenges include identifying inhibitors that bind to their targets taking into account differences in the conformation of inhibitor-bound, protein-bound, and unbound proteins.

The use of biochemical assays such as Protein Thermal Shift, NMR, and HTRF for demonstrating small molecule blocking of PD-1-PD-L1, PD-1-PD-L2, and PD-L1-CD80 interaction has been well established. On the other hand, there is paucity of established cell-based assays for verifying small molecule binding to PD-1 or PD-L1 and concomitant increase in functionality of PD-1-expressing exhausted T cells. ELISA-based binding assays and cell-based assays based on luciferase activity coupled to IL-2 production or nuclear factor and activator of T cells (NFAT) activity have been successfully used for testing anti-PD-1 monoclonal antibodies. Modification of these assays for demonstrating small molecule inhibitor binding may be accomplished by optimizing the concentration that will achieve PD-1/PD-L1 binding comparable to blocking antibodies. Downstream effects of PD-1 engagement to its ligands such as SHP2 phosphorylation and subsequent downregulation of PI3K activity (227) may serve as read-outs of inhibitor activity.

Additional factors to consider in the design and selection of small molecule inhibitors include toxicity (*in vitro*—for cell-based assays; *in vivo*—for animal studies), off-target interactions, and potency.

## Discussion and Concluding Remarks

**Figure 5:**
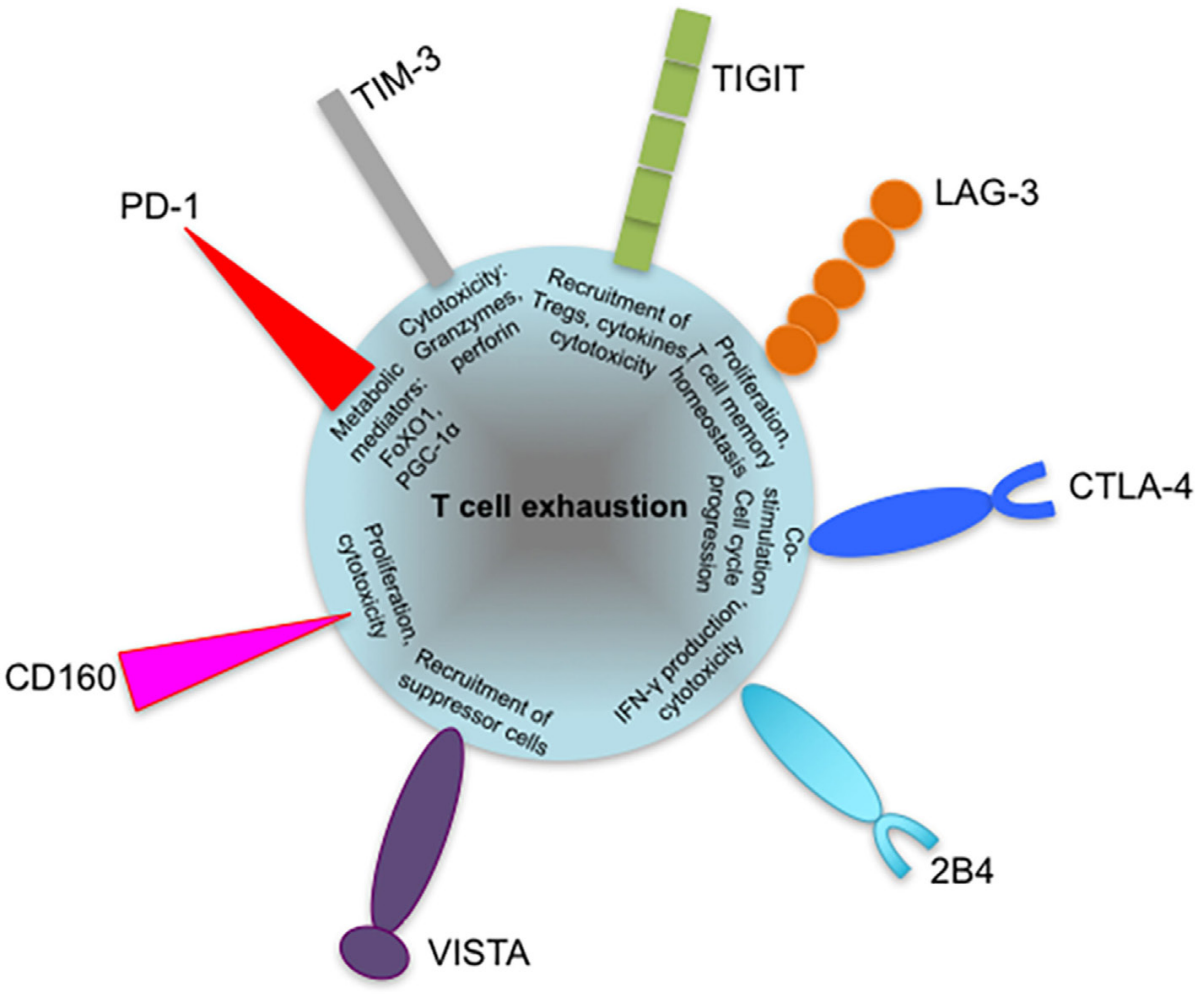
Coinhibitory receptors may play specific roles in regulating exhausted T cell responses. Severity of exhaustion has been shown to directly correlate with coexpression of multiple coinhibitory receptors on T cells. The diagram shows suggested roles of programmed cell death 1 (PD-1), T-cell immunoglobulin and mucin-domain containing-3 (TIM-3), lymphocyte activation gene 3 (LAG-3), cytotoxic T lymphocyte antigen-4 (CTLA-4), and T cell immunoreceptor with immunoglobulin and immunoreceptor tyrosine-based inhibitory motif domains (TIGIT) in contributing to T cell exhaustion, such as defective metabolism and cytotoxicity. The roles of other inhibitory receptors such as VISTA and 2B4 are yet to be identified. Coexpressed inhibitory receptors have been shown to synergize and increase the severity of T cell exhaustion. VISTA, V-domain immunoglobulin (Ig)-containing suppressor of T cell activation.

Mechanisms and factors that govern CD8^+^ and CD4^+^ T cell exhaustion are still being identified (Figure 2). Exhaustion of T cells is now regarded as a differentiation stage (132), arising in response to continuous TCR signaling during chronic infections or tumor development (50). Like other activated T cells, exhausted T cells portray various characteristics, now known to be driven by metabolic changes, ranging from reduced cytokine production, cytotoxicity, proliferation, altered, yet defective memory cell generation and upregulation of multiple inhibitory receptors. The heterogeneity of inhibitory receptors on the basis of their structure, level of cell expression, and nature of their ligands contribute to the non-redundant and synergistic nature of T cell inhibition. Due to constancy of expression in different exhaustion scenarios and the effectiveness of antibody blockade, PD-1 is regarded as the archetypal hallmark of exhaustion. However, identifying whether other inhibitory receptors such as CTLA-4, TIM-3, LAG-3, TIGIT, and VISTA play a global or specific role in regulating T cell function during exhaustion will improve current understanding of this phenomenon. Indeed results from chronic virus infections such as hepatitis and HIV indicate that expression of other coinhibitory receptors by responding CD8^+^ T cells may reflect their effector status and memory-differentiation potential (37, 70, 228). Observations from HIV studies indicate a central role for TIM-3 in influencing the cytotoxicity of HIV-specific CD8^+^ T cells. It is likely that certain inhibitory receptors, perhaps in conjunction with transcription factors such as GATA3 and NFAT (48, 229), type I interferons (230, 231), and regulatory TNF receptors are responsible for modulating various aspects of effector T cell metabolism, functionality and memory cell generation (Figure 5). This will explain the necessity for synergistic immune control facilitated by inhibitory receptors in response to prolonged antigen stimulation.

The suppressive tumor environment seen in cancers compared to highly inflammatory tissue sites in chronic virus infections is likely to contribute to differences in exhausted CD8^+^ T cells from both scenarios. PD-L1-expressing tumors and subsequent suppression of PD-1 expressed by CD8^+^ TILs has the potential to promote compensatory upregulation of other inhibitory receptor pathways. This occurrence in conjunction with the activity of myeloid derived suppressor cells leads to further T cell dysfunction. In addition, the nature of exhausted CD8^+^ T cells in cancers versus chronic virus infections is highly dependent on disease type or anatomical location. Studies now show that infections and tumors can dictate differentiation of T cell subsets and preferential upregulation of inhibitory receptors (91, 99, 232, 233).

Despite general upregulation of PD-1 and various combinations of cytokine loss and defective cytotoxicity by peripheral and tissue-associated T cells during chronic infections and cancers, a specific exhaustion signature is yet to be defined. The identification of biomarkers and cancer genetic markers will facilitate the development of novel diagnostic tests and treatment regimens. miRNAs such as miR-122, (234–237) and miR-200c (238, 239) have been identified as a biomarkers of HCV and cancer, respectively. Recently, miR-31, induced by calcium signaling has been shown to promote T cell exhaustion in chronic LCMV (240). Transcriptional profiling of exhausted T cells obtained from the periphery, tissues, and the tumor microenvironment will lead to the identification of novel and known miRNAs and genes that can serve as T cell exhaustion biomarkers.

The ability of metabolic changes in response to chronic TCR signaling to influence the exhaustion phenotype of responding T cells long before the manifestation of functional defects should serve as a determinant of future checkpoint blockade strategies. For instance, the ability of the glucose-depleted tumor microenvironment to compromise antitumor immune responses irrespective of antigenicity may influence the outcome to checkpoint blockade. Approaches that can restore CD8^+^ T cell mitochondrial health such as overexpression of PGC1α may be required before targeting inhibitory receptors such as PD-1 (241).

### Life after Exhaustion: Characteristics of Restored T Cells

A number of recent studies have focused on investigating the differentiation and characteristics of CD8^+^ T cells that constitute the “proliferative burst” after PD-1 checkpoint blockade and restoration of immune function (242, 243). Such cells have been defined based on expression of the transcription factor Tcf1, which is required for maintenance of protective immunity after antigen contraction in acute infections. Recent findings based on epigenetic stability of exhausted CD8^+^ T cells indicate that only partially exhausted CD8^+^ T cells can constitute or contribute to the differentiation of the proliferative burst (37, 70, 228). Importantly these cells have properties necessary for controlling persistent antigen: strong proliferative capacity characteristic of central memory cells, residence or ability to migrate to lymphoid organs. These observations have implications for other immune checkpoint strategies particularly coblockade. Studies on the metabolic requirements of these cells will further define the role of these cells in controlling chronicity. Furthermore, investigating whether maintenance of “exhausted” characteristics by these cells compromises their effector function in response to subsequent therapeutic approaches is essential. This will pave the way for the improvement and development of current and novel immunotherapy strategies, respectively. However, the way forward for success in immunotherapy lies in our ability to better understand human immune responses in the tumor microenvironment.

## Author Contributions

IO wrote the first draft of the manuscript. MH, LT, and KB provided comments, suggestions, and edited the manuscript. SE proposed the idea, wrote some sections, and edited the manuscript.

## Conflict of Interest Statement

The authors declare that the research was conducted in the absence of any commercial or financial relationships that could be construed as a potential conflict of interest.

## Abbreviations

CTLs, cytotoxic T lymphocytes; CTLA-4, cytotoxic T lymphocyte antigen-4; Gal-9, galectin 9; Gp, glycoprotein; irAEs, immune-related adverse events; ITIM, immunoreceptor tyrosine-based inhibitory motif; LAG-3, lymphocyte activation gene 3; LCMV, lymphocytic choriomeningitis virus; mTOR, mechanistic target of rapamycin; PD-1, programmed cell death 1; PD-L1, programmed cell death ligand 1; SHP, Src-homology domain-containing phosphatase; TIGIT, T cell immunoreceptor with Ig and ITIM domains; TILNs, tumor-infiltrated lymph nodes; TIM-3, T cell immunoglobulin and mucin-domain containing-3; VISTA, V-domain immunoglobulin (Ig)-containing suppressor of T cell activation.
